# Microneurosurgical treatment under the guidance of neuroendoscopy for an intracranial optic nerve Schwannoma: A case report

**DOI:** 10.1097/MD.0000000000020747

**Published:** 2020-06-19

**Authors:** Jin Xiao, Lei Ye, Weihong Wang, Hongwei Cheng, Changyuan Li, Jing Luo, Xiangyang Hu, Yiquan Zhang

**Affiliations:** a Department of Neurosurgery; b Department of Pathology, the First Affiliated Hospital of Anhui Medical University, Hefei, Anhui, PR China.

**Keywords:** neuroendoscopy, optic nerve, schwannoma, vasculature

## Abstract

**Rationale:**

Optic Schwannoma is rarely observed clinically as optic nerve had anatomically impossibility for the location of Schwannoma. However, several reports described the established cases of optic Schwannoma, of which the locations were in orbit or within optic canal. The occurrence of optic Schwannoma intracranially has been not reported.

**Patient Concerns:**

A 60-year-old female complained of visual impairment in the right eye and the frequent headache and the dizziness over 2 years with unknown reasons. The result of magnetic resonance imaging (MRI) showed a round, well-circumscribed, heterogeneously enhancing signal with cystic change displayed on the right suprasellar cistern.

**Diagnoses:**

Intracranial optic Schwannomas.

**Interventions:**

The patient accepted microneurosurgery assisted by endoscopy. We observed a gray and yellow lesion located near the right anterior clinoid process with a mid-sized cyst. And there was a vague boundary between the tumor and the right optic nerve which was compressed by the tumor. Optic chiasm and left optic nerve were also compressed. Meanwhile, the tumor had also adhesion to the right anterior cerebral artery (ACA).

**Outcomes:**

After the tumor was totally resected, the patient had satisfactory recovery.

**Lessons:**

We reported an intracranial optic Schwannoma removal with the lateral supraorbital keyhole approach assisted by neuroendoscopy. Intracranial optic Schwannoma was rarely seen clinically. Neuroendoscopy imaging suggested the close relationship between the tumor and ACA, supporting vasculature-origin hypothesis for the optic Schwannoma.

## 1 Introduction

Schwannoma is the primary benign tumor in peripheral nervous system arising from Schwann cells, accounting for 8% to 10% of all intracranial tumors. The most frequent location for Schwannoma is vestibulocochlear nerves, followed by trigeminal nerves.^[1]^ Optic nerve is not considered as a possible site for the origin of Schwannomas as optic nerve is ensheathed by oligodendrocytes rather than Schwann cells. However, there has been, in fact, several reports for optic Schwannoma.^[2]^ Two main hypotheses have been presented, including ectopic Schwann cell origin hypothesis and sympathetic nerve-related vasculature origin hypothesis.^[3,4]^

So far, a total of 8 reports of optic Schwannoma, including 12 cases, has been depicted.^[4–11]^ However, most of the tumors were located in the orbital region or within orbit canal. Here we reported a pure intracranial optic Schwannoma and we treated the tumor using microneurosurgical methods under the guidance of the endoscopy.

## 2 Case report

**Figure 1:**
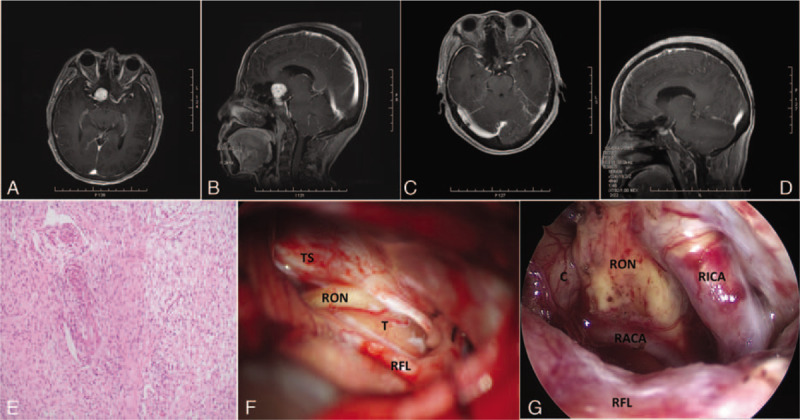
Magnetic resonance imaging (MRI) at pre-surgery (A and B) and post-surgery (C and D). Pathological examination confirmed the Schwannoma (E). Optic Schwannoma and surrounding structures under microscopy (F). Normal structures were demonstrated by 0^o^ neuroendoscopy after tumor removal (G). C = chiasm, RACA = right anterior cranial artery, RFL = right frontal lobe, RICA = right internal carotid artery, RON = right optic nerve, T = tumor, TS = tuberculum sellae.

A 60-year-old female presented to the neurosurgery department complaining of the visual impairment in the right eye accompanied with frequent headache and dizziness over 2 years with unknown reasons. She accepted magnetic resonance imaging (MRI) examinations, and a round, well-circumscribed, heterogeneously enhancing 22 × 17 mm mass with cystic change was displayed on the right suprasellar cistern. Right optic nerve was not shown clearly. The right anterior cerebral artery (ACA) was compressed by the lesion on magnetic resonance angiography (MRA) imaging which mimicked aneurysm (Fig. 1A and B). However, digital substraction angiography (DSA) was performed to exclude aneurysm and meanwhile to assess the blood supplies for the tumor. An evaluation by neuro-ophthalmology indicated normal visual field with lower visual acuity of right eye (0.3) than that of left eye (0.5). Both pupils were equal, round, and reactive to light and accommodation. Physical examinations in the neurosurgery clinic were unremarkable for any focal neurological findings.

Then, the patient accepted microneurosurgical treatment of the lateral supraorbital (LSO) craniotomy with the assistance of neuroendoscopy. After general anesthesia was administrated, the patient was positioned supine with shoulder and head elevated above the heart level. The head was fixed in a Mayfield headholder and rotated 15 degrees to the opposite side. An oblique frontotemporal incision behind the hairline was made. During the operation, we observed a gray and yellow lesion located near the right anterior clinoid process with a mid-sized cyst and there was a vague boundary between the tumor and the right optic nerve. The right optic nerve was compressed. Optic chiasm and left optic nerve were also compressed. Intratumoral decompress was performed in piecemeal manner followed by the release of the cystic fluid (Fig. 1 F and G). The tumor had heterogeneous texture and rich blood supply. When the tumor was nearly resected, we found a laminar tumor was closely adherent to the ACA, which was removed with sharp dissection carefully under 0 degree neuroendoscopy. Complete resection of the tumor was also identified by neuroendoscopy (Fig. 1C and D). Pathological examination confirmed the diagnosis of Schwannoma (Fig. 1E).

Sellar MRI with contrast revealed complete removal of the tumor at the 1-year follow-up. The patient's vision in the right eye was improved to 0.5 postoperatively. The symptoms for headache and dizziness also disappeared.

This study was approved by the Human Research Ethics Committee of Anhui Medical University and conducted within the confine of 1964 Helsinki declaration and its later amendments or comparable ethical standards. Patient has provided informed consent for publication of the case.

## 3 Discussion

Schwannoma originates from Schwann cells, with typical characteristics of slow growing, encapsulation, and mass effect on nearby structures. It is well known that optic nerve is myelinated by oligodendrocytes since their cell bodies arise centrally within the lateral geniculated nuclei.^[7]^ Therefore, there is no anatomic possibility for the origination of Schwannoma at the location of optic nerve. However, several reports have reported the orbital optic Schwannomas. The incidence of orbital Schwannoma is found to constitute 1% to 6.5% among all orbital tumors.^[12]^ Clinical presentations for those kinds of tumors usually include visual field loss, retro-orbital pain, insidious proptosis, and visual acuity hypofunction. In this report, we presented a case with pure intracranial Schwannoma with initial symptom of impaired vision in the right eye, accompanied by headache and dizziness. Operative exploration found the tumor located in the right anterior clinoid process, near optic chiasma, and had adhesive boundary with the right optic nerve. In comparison with previous reported orbital Schwannoma, the patient's symptoms were relatively mild. As both orbit and optic canal have incapacious space for the tumor growth, the patients whom the tumors located in those regions might present severer or more apparent clinical signs than those tumors of extraorbital locations.

As an extremely rare situation for optic Schwannoma, almost all the case reports discussed a valid question: does the lesion originate from optic nerve sheath itself? Several hypotheses might explain the reason. First, optic Schwannoma might originate from ectopic Schwann cells of the neural crest.^[1,13]^ Second, Schwannoma might arise from sympathetic nerves which are accompanied by Schwann cells and innervated by vasculatures of optic nerve and its sheath.^[14]^ However, radiological images and narrative descriptions just provided indirect evidences to support the hypothesis. In this case, we used endoscopy recording the detailed location and relationship between the Schwannoma and surrounding structures. The tumor had vague boundary with optic nerve, meanwhile was adhesive with ACA. This direct evidence supported the hypothesis of vasculature origin of Schwannoma. In addition, Civit et al^[15]^ observed that a intrasella Schwannoma had a dense attachment with dura, inferring that the tumor might be developed from Schwann cells converted either from mesenchymal cells with pluripotent differentiation capacity in the pia matter or from Schwann cells ensheathing the small nerve twigs innervating the dura. In this case, optic Schwannoma had no connections with dura.

The LSO approach by Hernesniemi et al^[16]^ for this case is a modification of the classical pterional approach by Yasargil et al.^[17]^ It is more subfrontal, less invasive, simpler, and faster. This kind of approach can be used for most pathologies involving sellar and suprasellar region as well as the tumors of the anterior skull base and sphenoid ridge. But it has the relative disadvantages such as poor illumination in the operative field, inconvenient hemostasis, and shadow of the local anatomic structure. The use of an endoscope can compensate for these deficiencies. With the assistance of endoscopy with different angles, it might provide close and almost full-scale observations and sufficient light sources when viewing intracranial lesions, especially for the deep lesions which the view of microscopy is difficult to reach.^[18]^ Meanwhile, the clear view from neuroendoscopy would also, in some extent, help us directly understand the anatomic information.

Intracranial optic Schwannoma reported here is a benign tumor just like the Schwannoma in the other locations. Preoperative MRI with contrast easily identifies the lesion. The applications of MRA or DSA could have differential diagnosis of the tumors which are adherent to the normal intracranial vessels from aneurysm. In previously reported cases, the symptoms which resulted from the Schwannoma could be alleviated by surgical treatments, such as the recoveries of vision and visual field^[4–9,11]^; however, the efficacy for a patient with the extremely large tumor size (8.5 × 5 × 5 cm) and a severe symptom of right eye proptosis was poor, with a sequela of visual loss.^[10]^ Total resection of the tumor may have a good prognosis. The efficacy about the radiotherapy in post-surgery is indefinite because of the lack of relative data support.

## Author Contributions

Conceptualization: JX and LY; Supervisor: HWC, CYL and YQZ; Investigation: WHW; Visualization: HWC and CYL; Methodology: JL; Writing-original draft: JX and LY; Writing-review & editing: XYH and YQZ.

## Author contributions

**Conceptualization:** Jin Xiao, Lei Ye.

**Investigation:** Weihong Wang.

**Methodology:** Jing Luo.

**Supervision:** Hongwei Cheng, Changyuan Li, Yiquan Zhang.

**Visualization:** Hongwei Cheng, Changyuan Li.

**Writing – original draft:** Jin Xiao, Lei Ye.

**Writing – review & editing:** Xiangyang Hu, Yiquan Zhang.
